# β2 Integrins—Multi-Functional Leukocyte Receptors in Health and Disease

**DOI:** 10.3390/ijms21041402

**Published:** 2020-02-19

**Authors:** Monika Bednarczyk, Henner Stege, Stephan Grabbe, Matthias Bros

**Affiliations:** Department of Dermatology, University Medical Center Mainz, Langenbeckstraße 1, 55131 Mainz, Germany; m.bednarczyk.09@aberdeen.ac.uk (M.B.); Henner.Stege@unimedizin-mainz.de (H.S.); stephan.grabbe@unimedizin-mainz.de (S.G.)

**Keywords:** β2 integrin, LFA-1, MAC-1, leukocytes, migration, phagocytosis, infection, LAD-I, cancer, autoimmune disease

## Abstract

β2 integrins are heterodimeric surface receptors composed of a variable α (CD11a-CD11d) and a constant β (CD18) subunit and are specifically expressed by leukocytes. The α subunit defines the individual functional properties of the corresponding β2 integrin, but all β2 integrins show functional overlap. They mediate adhesion to other cells and to components of the extracellular matrix (ECM), orchestrate uptake of extracellular material like complement-opsonized pathogens, control cytoskeletal organization, and modulate cell signaling. This review aims to delineate the tremendous role of β2 integrins for immune functions as exemplified by the phenotype of LAD-I (leukocyte adhesion deficiency 1) patients that suffer from strong recurrent infections. These immune defects have been largely attributed to impaired migratory and phagocytic properties of polymorphonuclear granulocytes. The molecular base for this inherited disease is a functional impairment of β2 integrins due to mutations within the CD18 gene. LAD-I patients are also predisposed for autoimmune diseases. In agreement, polymorphisms within the CD11b gene have been associated with autoimmunity. Consequently, β2 integrins have received growing interest as targets in the treatment of autoimmune diseases. Moreover, β2 integrin activity on leukocytes has been implicated in tumor development.

## 1. Introduction

**Figure 1:**
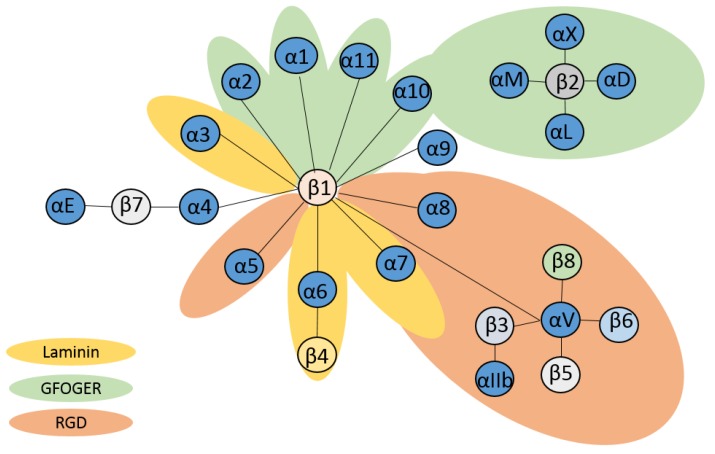
Representative members of integrins in vertebrates and their binding specificity.

Integrins are evolutionarily conserved heterodimeric transmembrane receptors that consist of an **α** and a **β** subunit [1]. Vertebrates possess a total of 24 **αβ** heterodimeric integrins, and some integrin receptors share the same **β** subunit. Integrins mediate intercellular as well as cell-matrix interactions [2]. Besides their role as adhesion molecules, some integrins also internalize extracellular material [3]. In general, binding of a ligand to an integrin induces signaling events that may result in cytoskeletal remodeling required, e.g., to confer cell migration [4], and activation/modulation of various signaling pathways [5]. As depicted in Figure 1, some integrins bind laminin, and/or the RGD peptide within their target ligands, whereas others recognize the triple-helical GFOGER sequence in collagens [2]. Some integrins also engage cell surface adhesion receptors (e.g., **β**2 and **β**7 integrins), apoptotic cells, and/or multiple other ligands. According to this classification, b1 and integrins as well as **α**V containing integrins constitute the three largest groups. **β**1α4, **β**1α9, all **β**2, and **β**7αE integrins are rather specifically expressed by leukocytes [6].

This review aims to highlight the role of **β**2 integrins in innate and adaptive immune cells since this group of receptors is crucially involved in leukocyte differentiation, activation/polarization, and functional activity. Therefore, impaired activity of **β**2 integrins results in dysregulated immune responses and severe diseases, including autoimmunity and tumor development [7]. Consequently, **β**2 integrins have been identified as highly interesting targets for therapy [6].

## 2. Structure and Activation of β2 Integrins

*β*2 integrins are heterdimeric surface receptors that are composed of one variable *α* subunit and a non-covalently bound constant *β*2 (CD18, gene name; Itgb2) subunit [6]. The *α* subunits are αL (CD11a, Itgal), αM (CD11b, Itgam), αX (CD11c, Itgax), and αD (CD11d, Itgad). *β*2 integrins dimerize intracellularly and are subsequently integrated in the cell membrane. Hence, single subunits are not detectable on the cell surface [8]. Since CD18 is expressed abundantly, the expression levels of the different *α* subunits limit the amount of the corresponding *β*2 integrin on the cell surface [9].

### 2.1. The α Subunit

**Figure 2:**
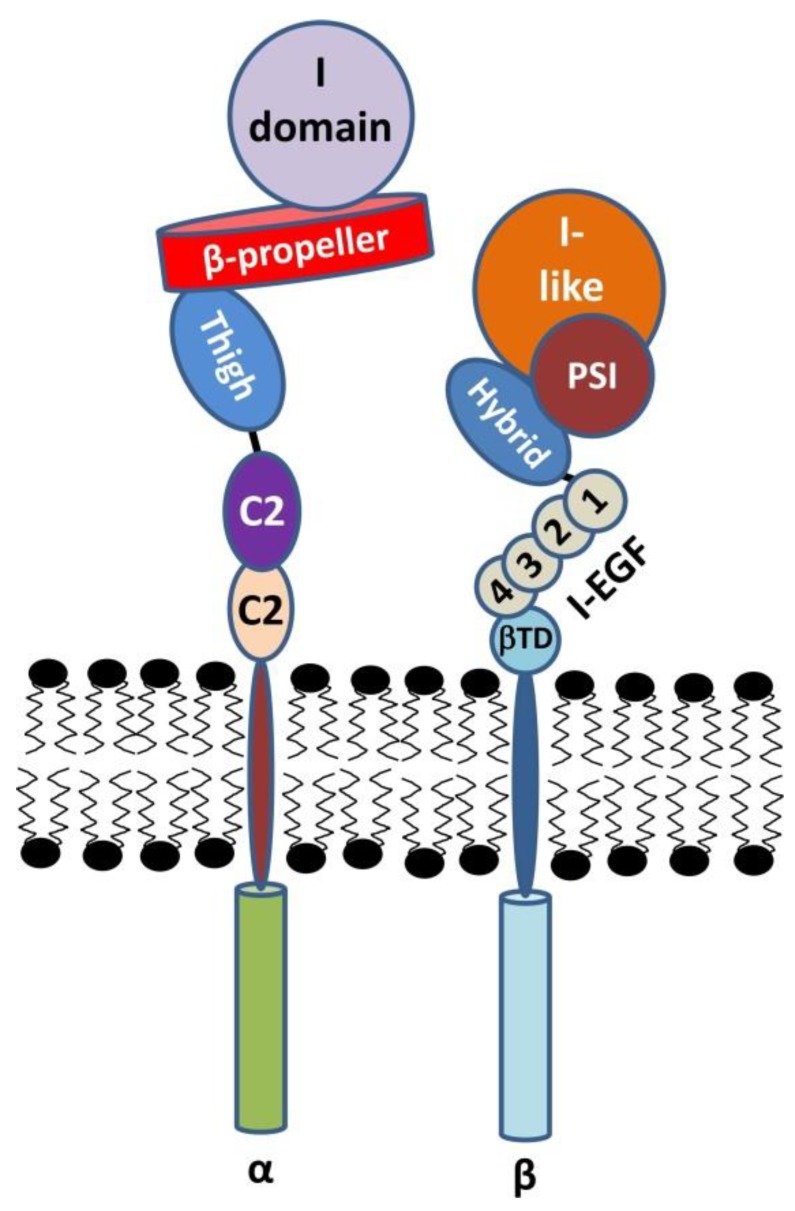
Structure of *β*2 integrins.

The head of any *α* subunit is composed of a seven-bladed *β* propeller motif that is connected via a thigh to the calf-1 (c1) and calf-2 (c2) domain (Figure 2). Calcium-binding EF-hand domains found within last three propeller blades promote ligand binding on the other pole of the propeller upon recruitment of a divalent cation [10]. Between the 2nd and 3rd blade of the *β* propeller a 200 amino acid I domain (also known as A domain) enables the *β*2 integrin receptor to bind collagen with its αC helix [11]. The *α*I domain, expanding the cleft between the *β* propeller and the *α*I domain of the *α* subunit, provides a binding surface that allows interaction with larger ligands. Binding of Mg^2+^ to the metal ion-dependent adhesion site (MIDAS) motif of the αI domain bridges binding of the *β*2 integrin to collagen or ICAM (intercellular adhesion molecule) over their negative glutamate-rich residues [12]. A C-terminal glutamate residue of the α1 domain constitutes an intrinsic ligand for the MIDAS on the β1 domain of the integrin β subunit, ensuring its conformational change upon ligand binding. The crystal structure of a metastable αXβ2 (CD11c/CD18) revealed that in a high affinity open form, α1α7 helices (C-terminal α7 of I domain) unwind and bind the *β* subunit between the *β* propeller and the *β*I domain [13]. Blockade of the interaction between the α1 and the β1 domains disrupts conformational changes [14]. Ligand binding to *β*2 integrin receptors may result in conformational alterations resulting in signal transmission into the cell, which has been termed outside-in signaling [3], conveyed by the *β*A domain of the *β* subunit. The C terminal portion of *α*l*α*7 reshapes into internal ligand and induces polar interaction between the *α* and *β* chains [15]. The functional role of the cytoplasmic tail of the *α* subunit is still unknown.

### 2.2. The β Subunit

The β subunit is connected to the cytoskeleton and conveys intracellular signaling. It contains eight extracellular domains: four epidermal growth factor (EGF)-like domains (EI-4), a so-called hybrid (H), a plexin-semaphorin-integrin (PSI), a *β* tail and a *β*I domain. The *β*I domain contains a MIDAS as well, which binds Mg^2+^ and thus bridges binding to aspartate residues within a ligand or to the glutamate residues within the αI domain. It acts as an allosteric regulator of αI-ligand binding [16]. Moreover, the intrinsic binding of Glu310 (αL) to the MIDAS of the activated βI domain leads to transmission of an activated state from the *α* to the *β* subunit, and is required for overall αM *β*2 activation [17]. Adjacent to the MIDAS, an ADMIDAS motif is located [18]. It constitutes a negative regulator inhibiting activation of the *β*2 integrin receptor at high Ca^2+^ concentrations and stabilizes its closed conformation. However, the ADMIDAS may also play a role in receptor activation as Mn^2+^ competes with Ca^2+^ for the binding to the ADMIDAS which results in receptor activation as demonstrated for β3 integrins [19]. The β1 domain contains a ligand-associated metal binding site (LIMBS) recruiting Ca^2+^ [20]. In case of *β*3 integrins, the LIMBS stabilizes binding of the metal ion at the MIDAS [21] and may play a similar role in case of *β*2 integrins.

### 2.3. Activation of β2 Integrins

**Figure 3:**
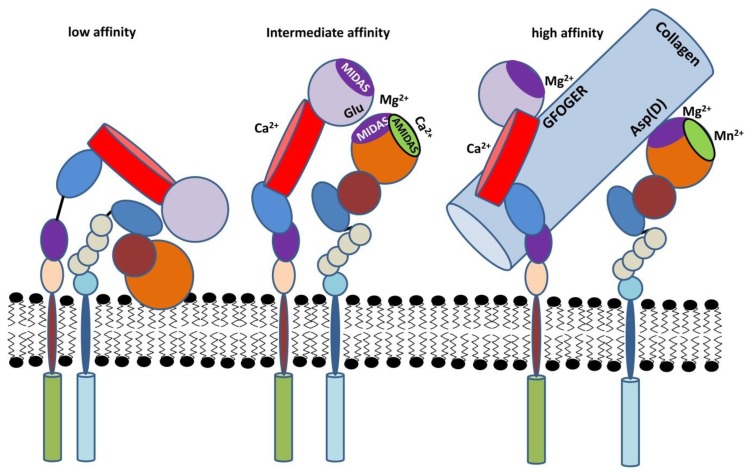
Conformational alterations determine the conformation and thereby the binding affinities of *β*2 integrins.

It is well known that integrins are normally expressed in a low affinity state on the cell surface with a bent closed headpiece (Figure 3). Activation of high-affinity binding and of intracellular signal transduction can occur via extracellular (`outside-in signaling´) or via intracellular signals (`inside-out signaling´) [22]. The inactive state of *β*2 integrins enables circulation of leukocytes in non-inflamed vessels [23]. As an exception, MAC-1 (macrophage-1 antigen, CD11b/CD18) on macrophages was reported to be in a high affinity state by default [24]. Engagement of an agonist (e.g., chemokines) and the presence of elevated levels of divalent cations result in inside-out signaling, and conformational changes of the extracellular domain of the integrin towards a more open, intermediate to high affinity state with an open headpiece. For the binding of some ligands, like ICAM-1, *β*2 integrins need to be in the high affinity state.

In comparison to LFA-1 (leukocyte factor 1, CD11a/CD18), inside-out signaling of MAC-1 is less well understood, and is distinctly regulated [25]. It is known for instance that stimulated MAC-1 orchestrates migration of polymorphonuclear granulocytes (PMN) in a MAPK-dependent manner, whereas activated LFA-1 induces phosphoinositide 3-kinase (PI3K)-dependent cell motility [26]. As outlined below, LFA-1-dependent cell migration depends *on the small GTPase Rap-1 which mediates inside-out signaling* [27].

**Figure 4:**
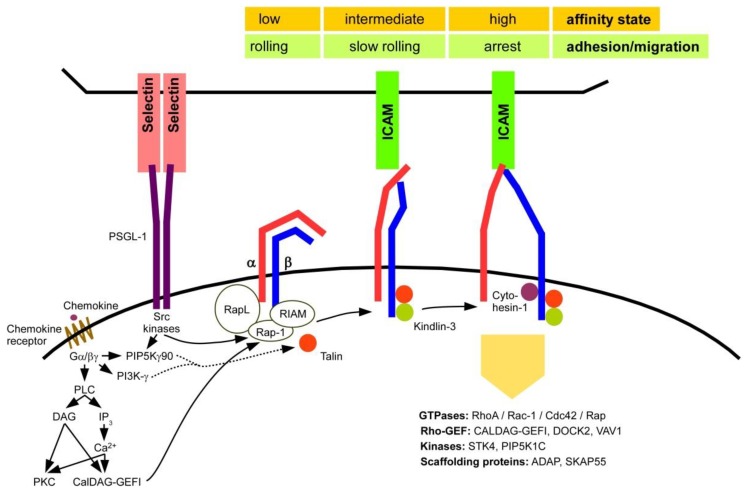
Regulation of *β*2 integrin inside-out signaling. Activation of signaling adaptor proteins and β2 integrin-binding proteins in response to engagement of chemokine receptors and PSGL-1 is indicated by arrows.

In response to chemokine binding, G*α*/G*βγ*-associated PLC (phospholipase C) generates the secondary messengers diacylglycerol (DAG) and IP3 (inositol trisphosphate) (Figure 4). In contrast to DAG, IP_3_ is soluble and confers release of Ca2^+^ from the endoplasmatic reticulum. DAG and Ca2^+^ activate PKC and CalDAG-GEFI (calcium and diacylglycerol-regulated guanine nucleotide exchange factor I) [28]. Further, Rap-1 activation is orchestrated by the regulator of adhesion and polarization enriched in lymphocytes (RAPL) molecule [29] which colocalizes both with active Rap-1 and CD11a. Downstream to RAPL the Ser/Thr kinase Mst1 (Macrophage-Stimulating Protein 1) subsequently binds the cytoplasmic CD11a domain and assures allocation of LFA-1 to the leading edge of the cell as a prerequisite for its migration [30]. RIAM (Rap-1-GTP interacting adaptor molecule) engages both active Rap-1 and CD18 [31] and mediates binding of Talin to CD18 [32]. Talin is necessary for unbending of the two *β*2 integrin subunits [33]. Kindlin-3 binds CD18 as well [34], and both Talin and Kindlin-3are required for the induction of a high affinity state of the *β*2 integrin [35]. Talin is necessary and sufficient to induce an intermediate state of *β*2 integrin affinity and to confer slow rolling of PMN on endothelium [36]. In contrast, in the same study both Talin and Kindlin-3 were reported as essential to confer a high affinity state and migration arrest to to binding of ICAM. More recently, Yago and co-workers reported that chemokine signaling also activated both PIP5K*γ*90 (phosphatidylinositol-4-phosphate 5-kinase *γ*90) and PI3K*γ* dependent signaling which cooperated with Rap-1 to achieve an intermediate state of affinity of LFA-1 [37]. In addition, binding of PSGL-1 (P-selectin glycoprotein ligand-1) to selectins as expressed by endothelial cells activated Rap-1 and PIP5K*γ*90 signaling via Src kinases as well. Besides Talin and Kindlin-3, also the Rho-GEF Cytohesin-1 was reported to bind CD18 and to be necessary for the high affinity state of LFA-1 [38], which as shown for DC required RhoA activation [39]. The importance of Kindlin-3 for *β*2 integrin activation and downstream functions is underscored by the observation that in human loss-of-function mutations of Kindlin-3 result in a LAD-I-like phenotype, termed LAD-III [40].

It has been shown that phosphorylation of serine residues near the C-terminus of the *α* subunit in both LFA-1 and MAC-1 is essential for receptor activation and thereby ligand affinity [41]. Subsequent to LFA-1 activation, the transcriptional activator JAB1 (Jun activating binding protein-1) was described to interact with the cytoplasmic portion of a cross-linked *β*2 integrin and to accumulate thereafter in the nucleus, where it triggers AP-1(activator protein-1) [42]. AP-1 is also a transcription factor and regulates differentiation, proliferation and apoptosis of the cell [43]. Chemokine-triggered *β*2 integrin activation can transduce signaling over small GTPases, Rap [27] and/or Rho (Ras homolog gene) [39], Rac-1 (Ras-related C3 botulinum toxin substrate 1) [44], Cdc42 (Cell division control protein 42 homolog) [45], according Rho-GEF such as, CALDAG–GEF [46], DOCK2 (Dedicator of cytokinesis 2) [47], VAV1 (Vav Guanine Nucleotide Exchange Factor 1) [48], kinases, including STK4 (Serine/threonine-protein kinase 4) and SKAP55 (Src kinase-associated phosphoprotein 5) [49], and other signaling proteins like PLD1 (Phospholipase D1) [50] and adaptor proteins as ADAP (Adhesion and degranulation-promoting adaptor protein) [49] and PIP5K1C (Phosphatidylinositol-4-Phosphate 5-Kinase Type 1 Gamma) [51,52]. Altogether, inside-out signaling induces a conformational change of the *β*2 integrin, leading to a high affinity open state, which results in strong binding of all ligands [53]. Engagement of a ligand conveys outside-in signaling resulting in cytoskeletal rearrangements.

### 2.4. Ligands of β2 Integrins

As mentioned above, the *α* subunit of a *β*2 integrin determines its ligand specificity. In this regard, activated LFA-1 engages ICAM 1-5, JAM (junctional adhesion molecule) 1, and ESM (endothelial cell-specific molecule) 1 [54]. MAC-1 binds numerous cell surface receptors at high affinity state and numerous soluble ligands irrespective of its state of activation. The former group comprises ICAM1-4, VCAM-1 (Vascular cell adhesion protein 1), JAM-3 (Junctional adhesion molecule 3), Thy-1 (Thymus cell antigen 1), RAGE (Receptor for advanced glycation endproducts), DC-SIGN (Dendritic cell-specific ICAM-3-grabbing non-integrin), and CD40L [41]. The list of MAC-1 binding soluble ligands is quite extensive, and includes fibrin(ogen), Factor Xa, platetelet Ib, heparin, polysaccharides, ssDNA, dsRNA, some acute phase proteins, HMGB1 (High mobility group box 1) and denatured proteins among many others as well as apoptotic bodies [55]. Moreover, MAC-1 binds several matrix proteins, like collagen, fibronectin, fibrinogen, vitronectin, Cyr61, and plasminogen. Further, MAC-1 is the primary receptor for complement C3-opsonized pathogens and immune complexes at high affinity, recognizing iC3b, C3dg, and C3d, and mediates their internalization [56,57]. Due to this property, MAC-1 has also been termed complement receptor 3 (CR3). (CR1 is widely expressed by leukocytes, and CR2 is predominantly apparent on B cells [58].) The spectrum of CD11c/CD18 ligands partially overlaps with that of MAC-1, including heparin, polysaccharides, and negatively charged denatured proteins [59]. CD11c/CD18 also binds ICAM1, ICAM4, Thy-1, and VCAM-1. Similar to MAC-1/CR3, CD11c/CD18 also recognizes complement-opsonized material, confers its uptake, and accordingly has been termed CR4 [56]. CD11d/CD18 bears structural similarity with MAC-1 and binds serum (Fibrinogen, Plasminogen) and ECM (Cyr61, Fibronectin, Vitronectin) components as well as cellular surface receptors (VCAM-1) [60] as previously reported for MAC-1. More recently, Yakubenko and co-workers demonstrated that PMN, as the first leukocyte population that infiltrates inflamed tissue, mark a path for subsequently attracted macrophages [61]. Activated PMN released myeloperoxidase (MPO), which in turn induced ROS (reactive oxygen species). ROS oxidized polyunsaturated fatty acids. Thereby, CEP (2-*ω*-carboxyethyl) are generated which modified proteins of the ECM. The derived CEP adducts were engaged at higher extent by MAC-1 and CD11d/CD18 than non-modified ECM components.

### 2.5. Expression Pattern of β2 Integrins

*β*2 integrins are expressed only by leukocytes. LFA-1 is rather ubiquitously expressed [62], whereas MAC-1 is predominantly apparent on myeloid cells, including PMN, monocytes/macrophages, and conventional dendritic cells (DC) [63]. In addition, it has been reported that MAC-1 is also present on the surface of NK (natural killer) cells and fractions of mast cells, B cells, CD8^+^ T cells, and CD4^+^ γδ T cells [64]. In mouse, CD11c/CD18 is rather specifically expressed by all DC populations, and accordingly serves as a well-accepted pan-DC marker [65]. Especially in human, CD11c/CD18 is also present on NK cells and lymphocyte subpopulations [66]. In mouse, CD11d/CD18 is expressed by a small fraction of leukocytes, being most abundant on myeloid cell lineages like macrophages and like MAC-1 is upregulated upon inflammation. In human, CD11d/CD18 is highly expressed on NK cells, B cells, and γδT cells as well [67].

## 3. Cellular Functions of *β*2 Integrins

**Figure 5:**
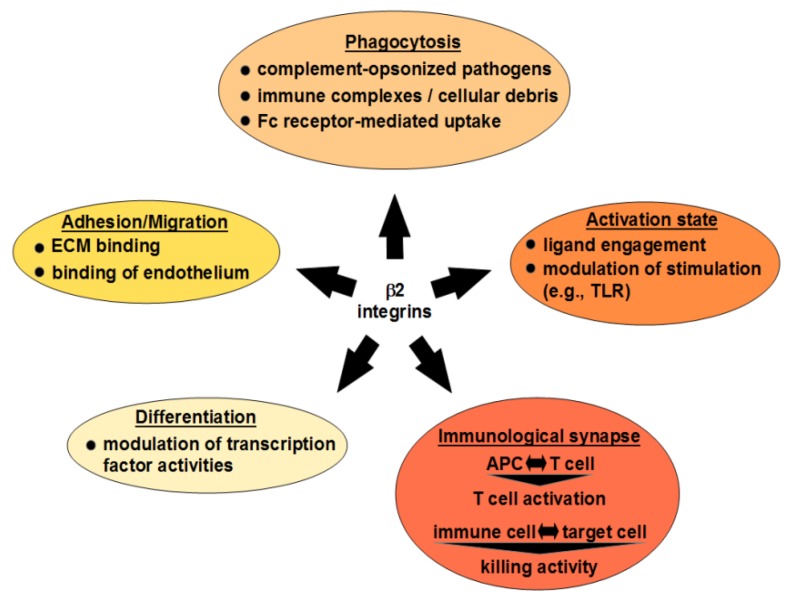
Schematic overview on the diverse functions of *β*2 integrins.

As outlined in the following, *β*2 integrins regulate the differentiation (3.1) and the functional properties of immune cells by exerting and modulating a large array of functions (Figure 5). In case of inflammation, *β*2 integrins confer cell migration, binding both components of the ECM as well as endothelial cells as a prerequisite tfiltrate inflamed tissue (3.2). *β*2 integrins play a dominant role for the uptake of opsonized pathogens and immune complexes (3.3). Further, *β*2 integrins regulate in part the state of cell activation in response to ligand engagement. In addition, *β*2 integrins also modulate the stimulatory activity of (other) danger signals comprising pathogen-derived molecular patterns like LPS [68] as well as endogenous mediators released in response to inflammation as for example TNF-*α* [69] (3.4). Moreover, *β*2 integrins regulate the extent and character of immune responses since they constitute essential components of the immunological synapse between antigen presenting cells (APC) and T cells (3.5.1) as well as between effector immune cells and infected/malignant target cells (3.5.2).

### 3.1. Cell Differentiation

LFA-1 contributes to the development of common lymphoid progenitors, resulting in lower numbers of thymocytes in CD11a^−/−^ mice [70]. However, in the periphery absolute numbers of T cell and B cells were affected only moderately, which suggested compensatory mechanisms. LFA-1 also regulates the polarization of CD4^+^ T cells. Both CD18^hypo^ [71] and CD11a^−/−^ [72] mice presented with diminished frequencies of CD4^+^CD25^+^ regulatory T cells (Treg), and these conferred diminished suppressive activity on stimulated naïve T cells. Moreover, when stimulated under Treg-promoting conditions, CD11a^−/−^ CD4^+^ T cells differentiated predominantly to pro-inflammatory Th17 cells. Preferred Th17 differentiation was also observed upon antibody-mediated blockade of CD18 in cocultures of DC with WT Treg [71]. Moreover, LFA-1 was reported as required for expression of the transcriptional silencer BCL6 (B cell lymphoma 6) [73] which in turn is a critical regulator of T follicular helper (Tfh) cells [74]. CD11a^−/−^ mice developed much less Tfh in response to infection than WT mice. In accordance with the role of Tfh to exert B cell help as necessary to induce humoral immune responses and to promote antibody affinity maturation [75], helminth-infected CD11a^−/−^ mice were characterized by diminished Tfh induction, and displayed an attenuated humoral immune response [73].

MAC-1 has been attributed an important role in the differentiation of bone remodeling cells. Bone homeostasis is mediated by the concerted activities of osteoblasts that generate, and osteoclasts that dissolve and absorb bone material [76]. Osteoclast progenitors express MAC-1, and macrophages can transdifferentiate to osteoclasts [77]. CD11b^−/−^ mice presented with bone loss that was associated with higher numbers of osteoclasts as compared with WT (wild type) animals [78]. MAC-1 expressing WT osteoclast progenitors were characterized by lower induction of the osteoclastogenesis transcription factor NFATc1 (nuclear factor of activated T cells, cxytoplasmic 1) in response to RANKL (receptor activator of nuclear factor kappa-Β ligand) than CD11b^−/−^ cells. MAC-1 mediated this effect by down-regulating the expression of RANK, and translocation of BCL6 to the NFATc1 gene promoter. Therefore, MAC-1 may constitute a negative feedback regulator of osteoclastogenesis. CD11d^−/−^ mice were characterized by reduced CD3 and CD28 expression by T cells, and an altered ratio of CD4^+^ and CD8^+^ T cells [79]. These alterations may be caused by a lack of CD11d/CD18 expression in the thymus resulting in inaccurate T cell development. The overall importance of *β*2 integrins for the expansion of HPSC (hematopoietic stem and progenitor cells) was demonstrated in a study by Meng and co-workers [80]. In a coculture system of HSPCs and Kupffer cells, that constitute the liver-resident macrophage population [81], Kuppfer cells promoted HSPC expansion and differentiation to lymphocytes. This was inhibited by antibody-mediated blockade of ICAM-1. Further, NK cells in CD18^−/−^ mice were characterized by partially inhibited differentiation as evidenced by an accumulation of c-kit^+^ progenitor cells, and NK hyporesponsiveness towards stimulation in vitro [82].

### 3.2. Migration

*β*2 integrins are essential for the recruitment of immune cells to sites of inflammation or tissue damage. All four *β*2 integrins bind proteins of the ECM, and surface receptors involved in intercellular interactions [83]. Therefore, *β*2 integrins enable a cell to adhere to the endothelium, and to extravasate blood vessels at inflamed sites [84]. As mentioned above, of all *β*2 integrins T cells express only LFA-1 [62], whereas myeloid cells (PMN, monocytes/macrophages and conventional DC) coexpress both LFA-1 and MAC-1 [63,64].

The relative importance of either of these two *β*2 integrins for migration of myeloid cells, especially of PMN, has been assessed in a number of studies. Heit and co-workers reported that MAC-1 and LFA-1 conferred chemotaxis of PMN in a ligand-specific manner [85]. While LFA-1 was required for IL-8-directed migration, MAC-1 was necessary for migration towards fLMP (N-Formyl-Met-Leu-Phe). Comparative analysis of CD11a^−/−^, CD11b^−/−^ and CD18^−/−^ PMN revealed that only WT and MAC-1-, but not LFA-1-deficient PMN engaged ICAM-1 at higher extent in response to stimulation with zymosan [86]. Intravital microscopy of leukocyte attachment to venules of muscles pretreated with TNF-*α* demonstrated that leukocyte velocities were highest in case of CD18^−/−^ mice in comparison to WT mice and displayed intermediate rates in case of CD11a^−/−^ and CD11b^−/−^ mice [87]. Leukocyte adhesion to TNF-*α*-stimulated endothelial was at comparably low level in case of CD18^−/−^ and CD11a^−/−^ mice and only somewhat lowered in case of CD11b^−/−^ mice. In a similar experimental approach, MAC-1 activity was identified as necessary and sufficient to confer rolling of PMN [88]. In contrast, rolling of monocytes was regulated by both LFA-1 and MAC-1 under non-inflamed conditions, whereas in response to treatment of muscles with TNF-*α*, MAC-1 played a more important role. In tissue, monocytes differentiate to macrophages that exert either proinflammatory functions to eradicate pathogens, termed M1 macrophages, or serve to dampen inflammation and to facilitate tissue healing (M2 macrophages) [89]. Recently, Cui and co-workers demonstrated that in vitro polarized M1 macrophages expressed CD11d/CD18 at much higher extent than M2 macrophages [90]. Elevated CD11d/CD18 expression enhanced the adhesion of M1 macrophages to the ECM and attenuated their migratory activity as compared to M2 macrophages both in vitro and in vivo as assessed by adoptive transfer of fluorescence-labeled macrophage populations in mice. Interestingly, in contrast to PMN and monocytes/macrophages, immature DC that engaged ICAM-2 on endothelial cells did not require *β*2 integrins for transendothelial migration [91]. The Rho-GEF Cytohesin—1 negatively regulates *β*2 integrin activity by engagement of CD18 [32]. Cytohesin-1 was reported to upregulate RhoA activity in stimulated DC, and both factors accounted for chemokine-induced *β*2 integrin activation [39]. In agreement, silencing of Cytohesin-1 impaired DC migration. CYTIP (cytohesin-1-interacting protein) sequesters Cytohesin-1 in the cytoplasm and thereby limits its interaction with β2 integrins [92,93]. Several viral pathogens were demonstrated to inhibit CYTIP in infected DC, which resulted in elevated LFA-1 activity, and thereby increased cell adhesion and impaired DC motility [94,95]. Furthermore, DC derived from mice with a knock-in of mutated CD18 containing a defective Kindlin-3 binding site showed a more mature phenotype and migrated at higher extent to draining lymph nodes both under steady state conditions [96] as well as after activation in a model of contact hypersensitivity [97].

In the course of T cell migration, redistribution of activated LFA-1 was reported to require Mst1 that is activated in response to chemokine stimulation and conferred activation of the GEF DENND1C (differentially expressed in normal and neoplastic cells domain 1C) as well as of the actin binding protein VASP1 (Vasodilator-stimulated phosphoprotein 1) [98]. Activated VASP1 mediated prolongation of actin filaments, while DENNDC1 activated the GTPase Rab13. Rab13 and Mst1 engaged LFA-1 and mediated its transport along VASP1-induced actin filaments engaging MyosinVa towards the front end of migrating T cells. Further, Mst1 also activated Myosin IIa which contributed to LFA-1 relocalization in migrating T cells as well [99]. Vesicular transport of LFA-1 from the rear to the cell front was reported to require activity of the small GTPase RhoB, which in turn activated Rab11 [100]. The latter is located in recycling endosomes and controls recycling of endocytosed proteins [101]. Moreover, T cell activation in the course of LFA-1 outside-in signaling activated PKC*ε* that mediated phosphorylation of the Rab GTPase Rab5a [102], which is primarily known as a constituent of endocytic vesicles [103]. Activated Rab5a relocalized to the front of migrating T cells and conferred Rac1 activation [102], known to be necessary for rearrangement of the cytoskeleton, and hence T cell migration [104]. The cystein protease Cathepsin X was demonstrated to negatively regulate the high-affinity state of LFA-1 by cleaving a minor part of the C-terminal end of LFA-1, which resulted in preferential binding of alpha-actinin-1 to LFA-1 [105]. Interaction of the PDZ-binding domain of the proteoglycan Syndecan-2 with LFA-1 was also reported to inhibit the acquisition of a high-affinity conformation and thereby elevated intercellular adhesion [106]. Triggering of plexin D1 by semaphorin 3E inhibited Rap-1, which in turn prevented LFA-1 activation and thereby impaired T cell migration [107]. In human monocytes, chemokine-induced LFA-1 activation was limited by the JAK family member PTPRG (protein tyrosine phosphatase receptor type g) [108].

### 3.3. Phagocytosis

MAC-1 was the first integrin receptor demonstrated to facilitate phagocytosis [109]. It plays a crucial role in the clearance of pathogens, tumor cells, apoptotic cells and of cellular debris that are opsonized with fragments of complement factor C3 [56]. Although physical interaction of MAC-1 with an FcR (Fc receptor) was never observed in murine immune cells, Jongsta-Bilen and colleagues (2003) demonstrated that in case of murine leukocytes that form a phagocytic cup upon FcR engagement MAC-1 accumulation was observed [110]. Likewise, as mentioned above CD11c/CD18 engages pathogens and other material opsonized with complement C4, and accordingly this β2 integrin receptor was also termed CR4 [57]. Hence, whereas FcR bind antibody-opsonized pathogens, MAC-1/CR3 and CR4 are the most important opsonophagocytic receptors of conventional DC. Moreover, in human PMN FcγRIIIB is constitutively associated with MAC-1 [111]. Similarly, MAC-1 was reported to physically interact with FcγRIIA in human PMN and to amplify calcium-mediated signaling of Fc*γ*RIIA/B, which resulted in an enhanced phagocytosis and release of pro-inflammatory cytokines [112]. The authors suggested that binding of immune complexes to the FcR resulted in intracellular rearrangements which in turn conferred release of MAC-1 from the cytoskeletal network and allowed its transfer to the site of phagocytosis [113]. There, MAC-1 could re-anchor to the cytoskeleton, and support FcR-mediated phagocytosis. Thus, several studies have confirmed that MAC-1, aside from its intrinsic phagocytic activity, is required for efficient FcR-mediated phagocytosis [114]. Consequently, MAC-1 may support the inflammatory process by amplifying FcR-mediated signaling, as well as by mediating migration of the leukocytes to the site of inflammation, ROS production, antibody-mediated phagocytosis, and release of pro-inflammatory cytokines. 

### 3.4. β2 Integrin Signaling Events in APC

*β*2 integrins have been demonstrated to modulate cell signaling in response to TLR (toll-like receptor) 4-mediated stimulation. CD14 is a surface membrane protein predominantly expressed by myeloid cell types which acts as a co-receptor for ligands of TLR2 and TLR4 but is also involved in uptake of inflammatory lipids [115]. CD14 is essential for LPS-triggered endocytosis [116]. CD11b was identified as a constituent of the receptor complex regulating TLR4 entry into bone marrow-derived DC [117]. CD11b deficiency resulted in reduced DC activation via TLR4 triggering by LPS which in turn attenuated the T cell stimulatory capacity of DC. Similarly, Perera and co-workers reported that synergistic activation of MAC-1, CD14, and TLR4 in murine macrophages is necessary for their responsiveness towards LPS [118]. Consequently, CD11b^−/−^ macrophages displayed diminished NF-*κ*B and MAPK signaling in response to LPS. In addition, Mac-1 was identified to serve as a receptor for dsRNA and to facilitate its internalization and thereby yielded enhanced TLR3-dependent signaling in macrophages [119]. Furthermore, internalized dsRNA induced NOX2 (NADPH Oxidase 2) in a TLR3-independent manner, which in turn activated both MAPK and NF-*κ*B.

On the contrary, several studies demonstrated that MAC-1 negatively regulated TLR-triggered inflammatory responses, preventing the onset of inflammation and subsequent tissue damage. In this regard, CD11b^−/−^ bone marrow-derived macrophages were shown to secrete higher levels of IL-6 and TNF-α in response to infection with Mycobaterium bovis Bacillus Calmette–Guerin (BCG) than WT macrophages [120]. DC recognize BCG via TLR2 and TLR4 [121]. Stimulation of TLR9 in CD11b^−/−^ DC yielded increased IL-12p70 production as compared with WT DC [122]. In agreement, Yee and Hamerman reported that CD18^−/−^ murine bone-marrow-derived macrophages and DC were hypersensitive towards various TLR ligands, as reflected by elevated production of IL-12 and IL-6 [123]. Bai and co-workers showed that MAC-1 inhibited TLR9- but not TLR4-induced expression of IL-12 in DC [122]. In case of TLR9 triggering, MAC-1 was required to induce miRNA-146a, which in turn inhibited expression of its genuine target NOTCH1 (neurogenic locus notch homolog protein 1), a well-known IL-12 inducer [124]. DC with a knock-in of CD18 mutated in the Kindlin-3 binding site and thereby inhibited *β*2 intgerin activity were demonstrated to contain higher levels of activated SYK (spleen tyrosine kinase), which via p38 MAPK activation conferred a more mature DC immuophenotype [96]. In agreement with the proposed anti-inflammatory activity of MAC-1 (under homeostatic conditions), engulfment of apoptotic bodies by human monocyte-derived DC via MAC-1 was reported to diminish their T cell stimulatory activity [125,126]. Moreover, MAC-1 was demonstrated to impair B cell receptor signaling in order to maintain autoreactive B cell tolerance [127] and dampened TLR3-dependent stimulation of NK cells [128].

Engagement of cytokines directly triggers the JAK (Janus kinase)/STAT (signal transducers and activators of transcription) signaling pathway [65]. As analyzed in a myeloid cell line, STAT3 activation in turn activated MAC-1, which resulted in homotypic cell aggregates [129]. Moreover, MAC-1 on human macrophages that bound ICAM-1 inhibited TLR signaling in an indirect manner by promoting expression of IL-10, SOCS (suppressor of cytokine signaling) 3, ABIN-3 (A20-binding inhibitor of NF-*κ*B activation 3), and A20 [97]. It has also been shown that pharmacological activation of MAC-1 on NK cells with leukadherin-1 reduced phosphorylation of STAT-5 in response to IL-12 stimulation and attenuated secretion of IFN- (interferon-) γ, TNF- (tumor necrosis factor-) *α*, and MIP1- (macrophage inflammatory protein 1-) β [130].

### 3.5. β2 Integrins in the Interaction of Immune Cells

#### 3.5.1. Interaction of APC and T Cells

##### Composition of the Immunological Synapse

The term immunological synapse (IS) designates the contact region between an APC and a T cell [131]. On cellular level, the IS structure has been studied most extensively for DC and CD4^+^ T cells [132]. The central supramolecular activation cluster (cSMAC) of an IS largely contains interacting receptor pairs of APC and T cells that confer antigen presentation (MHC, major histocompatibility complex) and recognition of the MHC/antigen complex by the TCR (T cell receptor) and TCR-associated coreceptors like CD3, CD4, and CD8 [133]. (Detailed graphical overviews of the IS structure, signaling processes initiated by T cell stimulation, and cytoskeletal rearrangements are given in [134] and [135].) In addition, the cSMAC contains receptor pairs required to transmit stimulatory signals from the APC (e.g., CD80, CD86) to antigen-specific T cells (CD28). The peripheral SMAC (pSMAC) among other receptor-ligand pairs is also characterized by a high density of ICAM-1 on the APC and its binding partner LFA-1 on the T cell side [136]. 

##### Role of LFA-1 in T Cell Activation

We showed that CD4^+^ T cells stimulated by DC proliferated at lower extent when lacking LFA-1 and were less prone to polarize towards Th1 [137]. This defect was rescued by additional antibody-mediated TCR stimulation, but not the costimulatory receptor CD28. These observations indicated that LFA-1/ICAM-1 interaction lowered the threshold required for optimal T cell stimulation. In agreement with the elevated threshold of TCR activation in LFA-1-deficient T cells, a normal dose of collagen sufficient to induce CIA (collagen-induced arthritis) in WT mice was insufficient in LFA-1^−/−^ animals [138]. However, a higher dose of antigen compensated for LFA-1 deficiency, inducing CIA. MHCII/antigen complex triggered TCR and ICAM-binding LFA-1 collaborated to trigger ERK-1/2 (Extracellular signal-regulated kinase-½) activation in T cells. Subsequent work showed that low affinity antigens depended at higher extent on LFA-1 than high affinity antigens to overcome the threshold of TCR activation [139]. As outlined above, LFA-1 activity is also required for Treg induction [71,72] and is a negative regulator of Th17 [71] and a positive regulator of Tfh [73] induction.

##### Regulation of LFA-1 activity on T Cells

Initial binding of MHC/antigen complexes to the TCR was shown to activate LFA-1 affinity by inside-out signaling [140]. Furthermore, an increasing number of adaptor molecules involved in the regulation of LFA-1 affinity and its spatial localization have been identified. Initial triggering of the TCR via the phosphatase SHP- (Src homology region 2 domain-containing phosphatase) 1 activated the adaptor protein CrkII and mediated its redistribution towards the pSMAC [141]. There, active CrkII recruited the GEF C3G, which activated the small GTPase Rap-1 (Ras-associated protein 1). Kondo and co-workers delineated that Rap-1 via Mst1/Mst2 activated NDR (Nuclear Dbf2-Related Kinase) 1 kinase [142]. In APC-binding T cells, Rap-1 activation was mediated in part by PI3Kδ [143]. Binding of semaphorin 3E to its receptor plexin D1 caused inhibition of the small GTPase Rap-1 [107]. This resulted in impaired activation of LFA-1, and attenuated IS formation. Activated NDR1 engaged Kindlin-3 and mediated its translocation towards the cSMAC [142]. The LFA-1 binding coactivator Kindlin-3 is defective in LAD-III patients [40]. T cells of a LAD-III patient in contrast to WT T cells showed no T cell spreading on surface-immobilized ICAM-1 when coincubated with DC [144]. Therefore, the authors concluded that Kindlin-3 transmitted TCR-triggered LFA-1 activation.

##### Cytoskeletal Rearrangements in the Course of T Cell Stimulation

A growing number of studies has highlighted the role of dynamic reorganization of the actin cytoskeleton in the context of IS formation and stabilization with the TCR and LFA-1 as important regulatory nodes [145]. Engagement of the TCR by MHC/antigen complexes induced F-actin relocalization from the cSMAC towards the pSMAC, yielding conformation-dependent activation of LFA-1. Binding of activated LFA-1 to ICAM-1 on the APC was observed to reduce the centripetal flow of F-actin from the cSMAC. Initial engagement of TCR was shown to result in TCR clustering which in turn caused recruitment of WASp (Wiskott-Aldrich syndrome protein) and Arp2/3 (actin-related protein-2/3) yielding actin polymerization [146]. Formins support actin polymerization by elongating actin filaments and inducing actin arcs within the IS [147]. Inhibition of Formins impaired TCR clustering within the cSMAC. TCR-associated Lck (Lymphocyte cell-specific protein tyrosine kinase) activates Crk-associated substrate lymphocyte-type (Cas-L), a force-sensing protein, which in turn mediated arrangement of TCR micro-clusters within the cSMAC [148]. In addition, activated CasL contributed to LFA-1 activation within the pSMAC. Caveolin-1 was required for spatial redistribution of LFA-1 towards the pSMAC [149]. Besides, the tyrosine phosphatase PRL-1 (phosphatase of regenerating liver 1) was demonstrated to be delivered aside with CD3ζ-containing vesicles towards the IS, and to colocalize with the TCR, especially CD3ε, and LFA-1 [150]. Pharmacological inhibition of PRL-1 affected actin rearrangements and IL-2 production.

LFA-1 is anchored to the underlying actin cytoskeleton by various proteins, including the actin-binding protein l-plastin in case of T cells [151]. Inhibition of L-plastin activity impaired LFA-1 redistribution, affected T cell/APC interaction, and resulted in attenuated T cell proliferation. Transgelin 2 (TGLN2) is predominantly expressed in T cells and served to stabilize cortical F-actin and at the same time engaged LFA-1 [152]. TGLN2 counteracted F-actin severing Cofilin activity. Consequently, TGLN2-deficient T cells formed unstable IS. LFA-1 colocalized with Formin-containing actin arcs, and Formin inhibition interfered with LFA-1 arrangement within the pSMAC, which caused attenuated IS stability and TCR signaling [147]. The cytoskeletal protein SPTAN1 (spectrin alpha, non-erythrocytic 1) also colocalized with LFA-1 in the course of IS formation [153]. siRNA-mediated SPTAN1 deficiency resulted in impaired adhesion of T cells to APC, and disturbed IS formation. Furthermore, the focal adhesion proteins paxilin, Talin, and vinculin colocalized with ICAM-1 engaging LFA-1 [154]. Of note, Talin and vinculin were required for inhibition of the centripetal F-actin flow as induced by TCR engagement, associated with attenuated tyrosine phosphorylation. Thereby, LFA-1, on one hand, is necessary for T cell/APC adhesion but, on the other hand, also dynamically regulates the extent/duration of TCR activation by modulating actin rearrangements within the IS [155].

##### β2 integrin Activity and Cytoskeletal Rearrangements on the APC Side

T cell priming also requires dynamic rearrangements of the actin cytoskeleton on the side of the interacting APC. WASp-deficient DC displayed diminished stability of the Arp2/3 actin filament network within the forming IS which resulted in lower accumulation of MHCII and ICAM-1 [156]. Rho signaling controls the activity of Cofilin that severs F-actin to enable dynamic cytoskeletal rearrangements [157]. We demonstrated that DC which lacked the Rho inhibitor Myosin IXB displayed strongly diminished Cofilin activity and contained less F-actin within the IS [158]. This was associated with altered DC/T cell contact duration and attenuated T cell proliferation within a 3D collagen micro-environment. The actin binding proteins moesin and α-actinin-1 bind the intracellular part of ICAM-1 and mediate its clustering within the IS to enable engagement of LFA-1 on the T cell side [159]. DC with mutated ICAM-1 lacking the cytoplasmic region initiated less IS and were poor T cell activators. Concerning the role of β2 integrins expressed by APC in the context of T cell activation, we reported for conventional DC that MAC-1 when activated by divalent cations attenuated CD4^+^ T cell proliferation [24]. In that study, we also demonstrated that inhibition of MAC-1 on macrophages using a blocking antibody enhanced their T cell stimulatory capacity. These findings confirmed the overall inhibitory activity of MAC-1 on APC. Furthermore, we showed that forced activation of LFA-1 on conventional DC by siRNA-mediated inhibition of LFA-1 binding CYTIP also increased CD4^+^ T cell stimulation [93]. DC with a non-functional Kindlin-3 binding site within the cytoplasmatic part of CD18 were characterized by elevated expression of surface activation markers and of IL-12 both at unstimulated state and after stimulation and consequently exerted stronger Th1 induction [96]. Altogether, these findings suggested that LFA-1 and MAC-1 on the DC surface may modulate the extent of adaptive immune responses. It is still unknown which counter-receptors on T cells are contacted by LFA-1/MAC-1 on the DC.

#### 3.5.2. Leukocyte/Target Cell Interaction

An IS also forms at the interface between two types of immune cells or between an (activated) immune cell and a target cell. Activated CD8^+^ cytotoxic T lymphocytes (CTL) were not able to stably adhere to an antigen-presenting tumor cells when CTL were pre-incubated with galectins [160] that bind cell surface glycans and are generated at high extent by tumor cells [161]. Impaired engagement of tumor cells by galectin-covered CTL was associated with impaired redistribution of LFA-1 to the CTL/tumor cell contact site. Antibody-mediated blockade of LFA-1 prevented the formation of such an IS as well. A decisive role of LFA-1 activity for CTL/tumor cell IS formation was confirmed by Wabnitz and co-workers who observed that oxidation-mediated hyperactivation of L-plastin which binds LFA-1 arrested the CTL/tumor cell contact [151]. This in turn attenuated overall CTL killing activity. Forced over-expression of the dual actin and LFA-1 binding protein TGLN2 in CTL yielded stronger killing activity in case of ICAM-1-expressing but not ICAM-deficient antigen-presenting tumor cells [162]. This observation suggested TGLN2-dependent inside-out activation of LFA-1. As mentioned above, TGLN2 was reported as well to stabilize F-actin at the pSMAC enabling LFA-1 mediated IS stabilization [152].

## 4. Pathophysiological Role of *β*2 Integrins in Human

Leukocyte adhesion deficiency (LAD) syndromes comprise a group of rare autosomal recessive disorders with an incidence of <1:10,000,000 (nearly 300 reported cases) [163]. In case of LAD-I, the molecular cause is impaired *β*2 integrin activity due to mutations within the CD18 gene [7,164]. LAD-I patients suffer from recurrent infections of bacterial and fungal origin, resulting in periodontitis, tooth loss, impaired wound-healing as well as severe leukocytosis, splenomegaly, and autoimmune symptoms [165,166]. In the severe form most LAD-I patients die before their 5th year of life, in a moderate form (5% to 15% residual CD18 activity) patients have a high chance of mortality between the 2nd and 4th life decade due to chronic infections [167,168]. CD18 deficiency described in dog and cattle presents with PMN dysfunction and recurrent infections of bacterial origin and thus resembles LAD symptoms reported for humans, which indicates that *β*2 integrin-associated pathology is conserved among mammalian species [169]. So far, two major blood cell types were considered to contribute largely to this phenotype, namely PMN and platelets, which are critical for the management of bacterial infections and rapid wound-healing, respectively.

The LAD-II syndrome, also known as congenital disorder of glycosylation type IIc, is characterized by symptoms similar to those noted in LAD-I, i.e., recurrent bacterial infections including pneumonia, periodontitis, and otitis media accompanied by leucocytosis [170]. The cause of LAD-II is a deficiency of the GDP-fucose transporter and consequently a defect in the synthesis of Sialyl-Lewis X, a P- and E-Selectin binding carbohydrate that is important for leukocyte tethering and rolling along the endothelium [60,171]. The LAD-III syndrome, also known as LAD-I variant’, is caused by mutations in FERMT3 (Fermitin Family Member 3)/Kindlin-3 that are important for the inside-out signaling and activation of *β*2 integrins. Thus, the adhesive function of leukocytes and platelets in LAD-III patients is impaired and cells cannot migrate efficiently [172,173,174]. So far, most research has focused on the role of PMN in LAD-associated pathologies. However, by now, other leukocyte populations were shown to play a crucial role in these maladies as well, especially in case of LAD-I and LAD-III.

## 5. Mouse Models to Study Functions of Distinct *β*2 Integrins

Even though LAD syndromes are quite rare, investigation of their pathomechanisms provides insight into fundamental immune processes and may serve to develop new immunomodulatory therapies. By now, several mouse models intended to reflect the LAD-I phenotype have been developed. CD18 knock-out mice have been generated first in the laboratory of A. L. Beaudet [175]. For this, an insertion mutation was introduced into the CD18 gene locus employing a homologous recombination approach in embryonic stem (ES) cells. Due to cryptic promoter activity exerted by targeting vector elements, low residual CD18 gene expression occurred which resulted in a hypomorphic CD18 allele (CD18^hypo^). The homozygous offspring displayed 2% to 16% of WT CD18 expression and was viable and fertile. CD18^hypo^ mice showed mild granulocytosis, an impaired inflammatory response to chemical peritonitis, and a delay in the rejection of cardiac transplants as well as erythrosquamous skin plaques that are strikingly reminiscent to psoriasis [176]. Thus, the CD18^hypo^ mouse model is well-suited to study psoriasis [177,178].

Subsequently, a full CD18 knockout mouse was generated [179]. The phenotype of CD18^−/−^ mice was much more severe as compared to CD18^hypo^ mice. About one third of CD18^−/−^ offspring died perinatally, and those that survived infancy developed extended facial and submandibular ulcerative dermatitis. Inflamed lesions contained lymphocytes but very few PMN, suggesting that migration of these cells was impaired. CD18^−/−^ mice developed granulocytosis, splenomegaly, and lymphadenopathy. They had about 10-fold increased serum IgG levels, and elevated IL-3 and IL-6 serum levels as compared with WT mice. The CD18^−/−^ mouse model resembles therefore a severe form of the LAD-I syndrome in humans and has been explored in the context of various pathologies like psoriasis [180], wound healing [181], diabetes [182], carditis [183,184], osteoporosis [185], and infections [186].

In order to assess the specific role of the different *β*2 integrins for immune functions, transgenic mice with constitutive deletion of either *α* subunit were generated. CD11a^−/−^ mice displayed mild neutrophilia and cleared bacterial infections inefficiently [187,188]. LFA-1-deficient mice failed as well to reject tumor xenografts and to respond to alloantigens due to the requirement of LFA-1 for IS formation and subsequent TCR signaling amplification [54]. In addition, LFA-1 was necessary for adhesion of T cells to infected and malignant cells and thereby for their killing [62]. CD11b^−/−^ mice were characterized by diminished PMN activation during inflammation and defective T cell proliferation in response to bacterial infection as a consequence of MAC-1 deficiency [189,190,191]. Interestingly, CD11b^−/−^ PMN were less prone to apoptosis [192]. Further, CD11b^−/−^ mice were more susceptible to develop autoimmune diseases [193]. In addition, CD11b^−/−^ mice were protected from thrombosis in response to injury as MAC-1 engagement of platelet GPIbα results in thrombus formation [194]. In some infection models CD11c^−/−^ mice showed an aggravated course of disease due to the requirement of CD11c/CD18 (CR4) for uptake of C4-opsonized pathogens for the induction of sustained adaptive immune responses [56,109,195,196]. CD11d-deficient mice in some cases displayed an aggravated course of infectious diseases [79].

Taken together, all four *β*2 integrins exert distinct functions, but display a functional overlap, such as migration and adhesion to inflamed endothelium. Consequently, the different *β*2 integrins can compensate for the lack of each other to some extent. None of the *β*2 integrin *α* chain (CD11a-CD11d) deficient mouse strains resembled the disease phenotype of CD18^hypo^ [175] and CD18^−/−^ [62] mice. In the following section, the pathophysiological role of *β*2 integrins for control of infections, the induction and course of autoimmune diseases and tumor progression, as well as therapeutic *β*2 integrin targeting approaches are discussed in more detail.

### 5.1. Functions of β2 Integrins in Infections

So far, the inability of the immune system of LAD-I patients to control infectious diseases has been largely attributed to functional defects of PMN [197] that constitute the first line of cellular innate host defense against pathogens [198] and of monocytes/macrophages [199]. As outlined above, β2 integrins essentially contribute to the immune functions of myeloid cells, comprising (i) chemokine-induced transendothelial migration due to engagement of endothelial ICAM by LFA-1 and MAC-1 [83]; (ii) recognition of complement-opsonized pathogens via MAC-1/CR3, and CR4 (CD11c/CD18) resulting in both cell activation and pathogen clearance via phagocytic uptake [56,200]; and (iii) cell activation by infection-induced cytokines or (pathogen-derived) ligands that engage MAC-1 and CD11d/CD18, which in turn initiates production of extracellular mediators that kill pathogens [200]. Besides, for canine PMN, LFA-1-mediated binding to ICAM-1 was shown to be sufficient to trigger release of hydrogen peroxide [201], which constitutes one of the various pathogen-killing mechanisms of myeloid cells [200]. In contrast to LFA-1-induced respiratory burst, MAC-1 mediated release of hydrogen peroxide, on one hand, required stimulation by chemokines but, on the other hand, induced much higher levels of hydrogen peroxide [201]. The role of β2 integrins in infections is outlined in the following.

#### 5.1.1. Viral Infections

In a HSV-1 (herpes simplex virus type 1) ocular infection model, CD11c^−/−^ mice showed lower virus titers than WT animals [202]. Attenuated viral loads were associated with stronger expression of IFNI (interferon type 1) and a higher frequency of virus antigen-specific CD8^+^ T cells in CD11c^−/−^ animals. So far, CR4 (CD11c/CD18) has not been associated with recognition/uptake of HSV-1 by DC, which is in agreement with the finding that HSV-1 has evolved strategies to counteract opsonization by complement [203]. It remains to be shown whether CD11c deficiency partially prevented infection of DC by HSV-1, and thereby HSV-1-induced inhibition of DC activation.

#### 5.1.2. Bacterial Infections

In a sepsis model CD11a^−/−^, mice were partially protected from lethal shock in response to administration of low doses of LPS [204]. The authors attributed the increased survival of CD11a^−/−^ mice to LPS-induced production of anti-inflammatory IL-10 by macrophages. This observation suggests that LFA-1 limits IL-10 production in myeloid cells. The role of LFA-1/ICAM-1 interaction in the context of infection-induced PMN infiltration was assessed in a model of aerosolized LPS application [205]. Here, PMN recruitment to the lung was roughly halved both in case of CD11a^−/−^ and ICAM-1^−/−^ mice. Similar effects were noted in WT mice when using blocking antibodies for either surface receptor. In a model of polymicrobial sepsis, lung infiltration of PMN was comparable in CD11b^−/−^ and WT mice [206]. However, CD11b^−/−^ mice were characterized by higher bacterial counts and stronger systemic inflammation, indicative of attenuated killing activity of MAC-1 deficient leukocytes. Mast cells have been reported to confer resistance towards acute septic peritonitis [207]. They were shown to express MAC-1 as well, and CD11b^−/−^ mice contained less mast cells as assessed in various organs [208]. Hence, the elevated mortality of CD11b^−/−^ mice in this disease model has been attributed in part to the reduced number and diminished functional activity of mast cells [207].

LAD-I patients with pneumonia displayed aggravated pulmonary PMN infiltration [209]. Similarly, Mizgerd and co-workers reported that infection of mice with *Streptococcus pneumonia* yielded strong PMN infiltration also in CD18^−/−^ mice [210]. In contrast, intratracheal administration of *Escherichia coli* and *Pseudomonas aeruginosa* resulted in strongly diminished pulmonary infiltration of PMN in CD18^−/−^ mice as compared with infected WT animals [211]. These findings suggest that β2 integrin deficiency may be compensated by other adhesion receptors in a disease-specific manner. Similar to CD18^−/−^ mice, CD11b^−/−^ mice infected with *S. pneumoniae* contained elevated numbers of pulmonary PMN as compared to infected WT mice but at the same time showed higher bacterial burden and stronger inflammation in lung than noted for WT mice. Again, these findings suggested functional impairment of CD11b^−/−^ leukocytes to kill pathogens. However, infection of CD11b^−/−^ mice with *Mycobacterium tuberculosis* yielded comparable bacterial burden, cytokine production, and development of granulomatous lesions as compared with WT animals [212].

As an immune evasive strategy, several pathogens like *Porphyromonas gingivalis*, the main inducer of periodontitis [213], bind CR3 [214] to prevent IL-12 induced pathogen-specific Th1 responses [215]. In agreement, CD11b^−/−^ mice infected with *P. gingivalis* contained higher serum levels of IL-12 and Th1-associated IFN-γ than infected WT mice. In accordance, CD11b^−/−^ mice cleared the pathogen more efficiently than WT animals [216]. In contrast, CD11a^−/−^ mice were characterized by periodontal bone loss [165] as also observed for LAD-I patients [166] as a consequence of infection with *Porphyromonas gingivalis*. Besides impaired infiltration of PMN required to control the infection, high levels of IL-17 were considered responsible for the aggravated course of periodontitis [165]. Antibody-mediated neutralization of IL-17 or the APC-derived Th17 promoting cytokine IL-23 attenuated the course of disease in CD11a^−/−^ mice. It is conceivable that the intrinsic property of LFA-1-deficient CD4^+^ T cells to differentiate towards Th17 may explain this phenotype in part.

*Listeria monocytogenes* is an intracellular pathogen which causes listeriosis in immunocompromised patients [217]. CD18^−/−^ [218] and CD11a^−/−^ [219] mice presented with higher survival after systemic administration of *L. monocytogenes* than observed for WT mice. This was associated with lower bacterial burden in liver and spleen and, accordingly, attenuated formation of necrotic lesions. In both knockout strains infection with *L. monocytogenes* caused elevated serum levels of G-CSF (granulocyte-colony stimulating factor) known to promote PMN differentiation [220]. *L. monocytogenes*-infected CD18^−/−^ mice also displayed enhanced serum contents of the innate proinflammatory cytokine IL-1β [218]. In likewise infected CD11a^−/−^ mice, numbers of liver-infiltrating PMN were elevated, and IL-17 serum levels were enhanced [219]. At early time points after infection with *L. monocytogenes*, liver PMN of CD11a^−/−^ mice generated highly increased levels of IL-12, which in turn induced IFN-γ production by NK cells [221]. In WT mice, antibody-mediated blockade of CD11b prior to infection with *L. monocytogenes* caused enhanced liver infiltration of PMN and clearance of the pathogen by PMN and Kupffer cells [222]. The extent of CD8^+^ T cells responses in mice deficient for α subunits of β2 integrins (CD11a-CD11c) in response to infection with *L. monocytogenes* was assessed in a comparative study [186]. Only in CD11a^−/−^ mice significant differences as compared with WT animals were observed. In these mice the primary CTL response was strongly attenuated, whereas the generation of central memory CD8^+^ T cells remained largely unaltered.

CD11c^−/−^ mice presented with an aggravated form of Lyme carditis upon infection with the spirochete *Borrelia burgdorferi*, which was most likely caused by an increased infiltration of macrophages [223]. Thus, CR4 (CD11c/CD18) may be important for the clearance of infections mediated by pathogens that are opsonized by C4 [56,109,195,196]. In a peritoneal *Salmonella Typhimurium* infection model, CD11d^−/−^ mice were characterized by diminished peritoneal infiltration of leukocytes and accordingly elevated bacterial loads [224]. Ex vivo analysis confirmed attenuated phagocytic activity of CD11d^−/−^ macrophages, accompanied by increased cell death in the course of the inflammatory response, termed pyroptosis [225].

#### 5.1.3. Fungal Infections

Immunocompromised human, including LAD-I patients, often suffer from pulmonary infections with *Aspergillus fumigatus* [226]. Recently, we showed that lungs of CD11b^−/−^ mice infected with *A. fumigatus* contained higher numbers of PMN than observed for WT animals [190]. However, lungs of infected CD11b^−/−^ mice presented with a higher fungal burden and contained less innate proinflammatory mediators than apparent in infected WT mice. In addition, CD11b^−/−^ PMN exerted lower phagocytic activity on complement-opsonized *A. fumigatus* conidiae. The latter finding is in agreement with a report of Gazendam and co-workers demonstrating that in human CR3-dependent phagocytotic uptake of complement-opsonized *A. fumigatus* conidiae is the primary mechanism for fungal killing [226]. However, *A. fumigatus*-triggered PMN activation also yielded release of NET (neutrophil extracellular trap) [227] that are largely composed of decondensed chromatin, and trap pathogens [228]. Clark and co-workers revealed that *A. fumigatus* cell extracts which stimulated PMN via CR3 also induced ROS production and ROS-dependent NETosis [229]. Like invasive pulmonary aspergillosis, invasive candidiasis frequently occurs in immunocompromised humans [230]. *Candida albicans* is cleared by CR3-mediated phagocytosis [200], NETosis [231] as well as ROS [232]. Hence, in a mouse model of candidiasis CD11b^−/−^ mice displayed attenuated PMN killing activity and increased fungal burdens [233].

#### 5.1.4. Metazoan Parasites

Leishmania are obligate intracellular unicellular parasites that enter macrophages and cause severe skin lesions [234]. In experimental leishmaniasis, footpads of CD18^−/−^ mice infected with *Leishmania major* were not infiltrated by PMN, in contrast to infected WT animals [235]. In vitro analysis revealed that CD18^−/−^ macrophages phagocytosed complement-opsonized *L. major* at lower extent than WT macrophages, which may be explained by lack of MAC-1/CR3. In that study, scavenger receptors that engage a broad range of extracellular compounds including pathogens [236] were shown to confer *L. major* uptake. Further, CD18^−/−^ macrophages were unable to generate NO [235] which contributes to *L. major* killing [237]. T cells derived from *L. major*-infected CD18^−/−^ mice poorly proliferated upon restimulation, which is in agreement with the pronounced role of LFA-1 for T cell activation [62]. Similar to other pathogens, *L. major* was shown to limit IL-12 production by APC via binding to MAC-1/CR3 [238]. In line, *L. major*-infected CD11b^−/−^ mice were characterized by a milder course of cutaneous leishmaniasis [239]. It is tempting to speculate that the lack of CR3 prevented inhibitory effects of Leishmania engagement on IL-12 production which is required to mount a Th1-biased anti-Leishmania immune response [240].

Infection of CD11d^−/−^ mice with the malaria pathogen *Plasmodium berghei* induced less systemic inflammation and attenuated lethality as compared to infected WT animals [241]. However, the advantage of survival of *P. berghei*-infected CD11d^−/−^ mice was not associated with differences in parasite load. Further, although Th1-biased immune responses are crucial for control of malaria in the acute phase [242], serum of *P. berghei*-infected CD11d^−/−^ mice contained less amounts of the Th1-promoting cytokine IL-12 than observed for infected WT animals [241]. Hence, the mechanisms that confer an attenuated course of malaria in CD11d^−/−^ mice are unclear so far. CD11d deficiency also attenuated MA-ARDS (malaria-associated acute respiratory distress syndrome) [243]. Infected CD11d^−/−^ mice displayed less lung infiltration by leukocytes, lung inflammation and airway hyper-responsiveness than infected WT animals.

Altogether, these findings suggest that with regard to migratory activity, loss of either LFA-1 or MAC-1 in myeloid cells may be compensated by the other *β*2 integrin [87]. In addition, the activity of *β*2 integrins as such may be dispensable in some types of infections, presumably due to partial compensation by other adhesion receptors [244,245,246]. Anyway, loss of MAC-1/CR3 on one hand may diminish pathogen clearance by phagocytic uptake [56] and also attenuate other killing mechanisms of myeloid cells [201,235]. However, a number of pathogens exploits MAC-1/CR3 induced inhibition of IL-12 production in APC and thereby sustained pathogen-specific Th1-biased immune responses [216]. Therefore, the net outcome of MAC-1/CR3 deficiency concerning the course of an infection may depend on the relative functional importance of this *β*2 integrin for innate and adaptive immunity. Besides, it is noteworthy that also engagement of LFA-1 by some pathogens results in impaired IL-12 production [221]. Furthermore, pathogens have developed additional immune evasion strategies based on modulation of *β*2 integrin activity. For example, infection of DC by HSV-1 [94] and cytomegalovirus [95] impaired their migratory activity due to virus-induced downregulation of CYTIP-1. This resulted in enhanced LFA-1 activity, and thereby increased cell adhesion. We have shown that active LFA-1 on DC impaired their T cell stimulatory capacity [93]. It is conceivable that lowered migration (to secondary lymphoid organs) and attenuated T cell activity of infected DC may cause impaired adaptive pathogen-specific immune responses.

### 5.2. Functions of β2 Integrins in Autoimmunity

A growing body of research suggests that *β*2 integrins play an important role in tolerance induction [247,248,249] and suppression of inflammation [83,250,251]. Already decades ago, it was noted that LAD-I patients suffer not only from bacterial infections but also from renal or intestinal autoimmune diseases [252,253], and some of them presented type 1 diabetes or autoimmune cytopenia after hematopoietic stem cell transplantation [254]. Likewise, CD18^−/−^ mice are characterized by chronic dermatitis and splenomegaly, which indicates a circulating inflammation [179]. These findings may be explained by a tolerance-promoting role of *β*2 integrins. As outlined above, LFA-1 is required for the differentiation and suppressive activity of Treg, and its deficiency actually promoted induction of proinflammatory Th17 cells [71,72]. Furthermore, MAC-1 was shown to be required for the induction of peripheral tolerance by suppressing IL-6 secretion and subsequent Th17 differentiation in a model of orally induced tolerance in mouse [255]. Furthermore, MAC-1 maintained autoreactive B cell tolerance by inhibiting B cell receptor signaling [127]. Besides, we have reported that activated LFA-1 on DC [93] and MAC-1 on DC and macrophages [24] limited their T cell stimulatory capacity. Most studies have addressed the role of LFA-1 and MAC-1 for the onset and course of autoimmune diseases in various mouse models. The main observations of according studies are summarized in Table 1.

#### 5.2.1. LFA-1

LFA-1 plays a crucial role in transendothelial migration of activated T cells and stabilizes the APC/T cell contact, thereby strengthening TCR signaling [256]. Inducible EAE (experimental autoimmune encephalomyelitis) is a well-established mouse model that recapitulates many aspects of multiple sclerosis (MS) [257]. For induction of EAE, mice are immunized with a protein of the neuronal myelin sheath, e.g., MOG (Myelin oligodendrocyte glycoprotein), in combination with an adjuvant. Both in MS and EAE the autoinflammatory phenotype is mediated predominantly by CD4^+^ Th17 (T helper cell type 17) cells. EAE can also be induced by transfer of CD4^+^ T cells from a mouse that has developed EAE (encephalitogenic T cells) to a naive one. CD11a^−/−^ mice presented with a reduced frequency of immuno-suppressive Treg fraction in the CNS, which resulted in more severe EAE after immunization [258]. Likewise, CD18 expression was found to be necessary for Treg induction [259,260]. Thus, on one hand transfer of encephalogeneic WT T cells to CD11a^−/−^ mice—lacking Treg—caused a fatal course of the EAE [261]. On the other hand, transfer of encephalogeneic T cells from CD11a^−/−^ mice, immunized to develop EAE, to WT mice resulted in an attenuated course of EAE, which confirms that T cells require LFA-1 to get primed and to mediate an inflammatory response [261,262].

Concerning the role of LFA-1 in other autoimmune manifestations in humans, an elevated expression of LFA-1 on T cells was shown to correlate with the severity of systemic sclerosis, systemic lupus erythematosus (SLE) [263], rheumatoid arthritis (RA), and autoimmune thrombocytopenia [264,265]. In order to suppress T cell priming and effector functions, antibody-mediated blockade of CD11a was successfully used in treatment of psoriasis vulgaris [266]. CD11a blockade using Efalizumab resulted in an unexpected downregulation of a broad range of T cell surface molecules including the TCR, costimulatory molecules and integrins unrelated to LFA-1, both in the peripheral circulation and in diseased skin. Unfortunately, Efalizumab had to be withdrawn from the market since it caused reactivation of the JC virus and subsequent progressive multifocal leukoencephalopathy in some patients [267,268].

#### 5.2.2. MAC-1

In CD11b^−/−^ mice, the onset of EAE was delayed, and the course of disease was less severe as compared to WT animals [269]. In accordance, CD4^+^ T cells derived from immunized CD11b^−/−^ mice were characterized by elevated production of anti-inflammatory (e.g., TGF-*β*, IL-10) and attenuated generation of pro-inflammatory (e.g., IFN-*γ*, TNF-*α*) cytokines as compared to WT T cells. In agreement, adoptive transfer of encephalogeneic T cells from CD11b^−/−^ mice to WT mice induced no EAE. Vice versa, transfer of encephalogeneic T cells from WT to CD11b^−/−^ mice resulted in a mild course of disease. Taken together, these findings suggest that in EAE MAC-1 is required to induce a profound inflammatory response. Moreover, CD11b^+^ B cells were demonstrated to suppress TCR signaling in a mouse model of experimental autoimmune hepatitis [189].

In a murine collagen-induced arthritis (CIA) model, MAC-1 expression prevented early disease onset, and decreased the severity of CIA via controlling IL-6 secretion and subsequent Th17 polarization of lymphocytes [255]. On C57BL6 genetic background, CD11b^−/−^ mice developed CIA of high severity and incidence, whereas WT mice were fully protected from the disease. [255]. Adoptive transfer of WT DC to CD11b^−/−^ animals reduced the severity of arthritis. As mentioned above, MAC-1 controls in part the phagocytic activity of FcR [114]. In a humanized mouse model with leukocytes expressing human FcγRIIA, CD11b deficiency protected from lupus nephritis that normally develops in response to injection of human SLE sera [270]. The role of MAC-1 for PMN-mediated autoimmune diseases was assessed in a model of Fc-dependent anti-GBM (glomerular basement membrane) nephritis [271]. In this model, application of anti-GBM antibody results in glomerular PMN accumulation due to binding/uptake of GBM/antibody immune complexes via FcR. In CD11b^−/−^ mice, PMN infiltrated glomeruli initially at similar extent as observed for WT animals, but later on at much lower rate [272]. In agreement, CD11b^−/−^ mice developed no proteinuria, in contrast to WT mice. Ex vivo analysis of PMN showed that MAC-1 was not required for PMN migration and adhesion but that interaction of MAC-1 and FcR was necessary for for F-actin rearrangements leading to long-lasting PMN adhesion. Bullous pemphigoid (BP) is an IgG-mediated autoimmune disease resulting in skin blisters [273]. In mice, BP is induced by injection of the hemidesmosome antigen BP180 mediating accumulation of CD11b^+^ cells in the skin that confer inflammatory reactions [274]. In CD11b^−/−^ mice the development of BP in mice was significantly lower as compared with WT mice, since CD11b^−/−^ PMN could not efficiently infiltrate the skin [275].

In agreement with the role of MAC-1 to contribute to the induction and maintenance of tolerancein, human CD11b gene polymorphismus which inactivated CD11b were shown to be associated with development of SLE and RA [276]. Three common CD11b SNP (single-nucleotide polymorphisms) translate into miss-sense mutations (P1146S, R77H, A858V), which are associated with SLE pathogenesis [277] and its common complication, lupus nephritis [278].

#### 5.2.3. Other β2 Integrins

Compared to LFA-1 and MAC-1, the role of CD11c/CD18 (CR4) and CD11d/CD18 for the induction and progression of autoimmune diseases has been studied less intensively. Concerning CD11c, it was shown that the iC3b binding site of CR4 was required for the induction of the delayed type hypersensitivity [279,280]. Aziz and co-workers reported that M1 macrophages and white adipose tissue upregulated the expression of the CD11d, which in turn may contribute to the chronification of inflammation [67]. Furthermore, it has been noted that antibody-mediated activation of CD11d/CD18 increased IL-1*β* expression [281], known as strongly associated with acute and chronic inflammation [282].

To sum up, all four β2 integrins contribute to inflammation. LFA-1 and MAC-1 have been extensively studied in various rodent autoimmune models like EAE, SLE, and CIA and contribute to (auto)inflammation by supporting cell migration, uptake of autoantigens, subsequent stimulatory cell signaling, and T cell activation.

### 5.3. β2 Integrins and Tumor Development

Whereas tumor infiltration by T cells that commonly express LFA-1 strongly correlates with overall prognosis and with response to checkpoint inhibitor-based immunotherapy in mice and men, tumor infiltration with myeloid cells is often associated with poor prognosis and rapid tumor growth [298]. In established solid tumors, tumor-infiltrating monocytes differentiate to tumor-associated macrophages (TAM) [299]. TAM primarily serve to promote tumor growth by various mechanisms, including the generation of angiogenetic factors like VEGF (vascular endothelial growth factor) and the release of immunomodulatory factors that counteract tumor-infiltrating immune effector cells [300]. Moreover, immunomodulatory cytokines as generated by TAM and tumor cells interfere with the differentiation of myeloid progenitor cells [301]. This results in the induction of monocytic (CD11b^+^Ly-6C^+^) and granulocytic (CD11b^+^Ly-6G^+^) myeloid-derived suppressor cells (MDSC) [302]. Similar to TAM, MDSC accumulate in the TME but also in lymphoid tissues exerting immunosuppressive functions by directly tolerizing APC and inhibiting effector T cells by various mechanisms [303]. In the following, the role of β2 integrins for tumor development are discussed, and major findings are summarized in Table 2.

#### 5.3.1. Tumor Infiltration

Infiltration of CD18^+^ cells early in tumor development was reported to prevent its progression. [304]. However, at later stages of tumor development infiltration of a tumor by myeloid cells may turn into a disadvantage, since TAM and MDSC that can induce tolerance localize to the tumor site and support its progression by various mechanisms [305,306,307]. Immunohistochemical analysis of human gastric tumor tissues revealed that most tumor infiltrating CD11b^+^ cells were (conventional) CD11c^+^ DC and that high infiltration of these cells correlated with tumor size, venous invasion, lymph node metastasis, the general metastasis stage, and infiltration by FoxP3^+^ Treg [308]. Accordingly, patients with high CD11b^+^ cell infiltration of the tumor had a poor outcome [281,307,308]. In agreement, Zhang and colleagues showed that CD11b deficiency in mice resulted in reduced infiltration of spontaneous intestinal adenoma with myeloid cells and attenuated tumor growth [305]. Systemic application of CD11b-specific monoclonal antibodies increased the anti-tumor response after radiation in mice, as myeloid cells could not migrate to the tumor site and support tumor angiogenesis (see below) [309].

#### 5.3.2. Tumor Angiogenesis

A study conveyed by Soloviev and colleagues has shown that not only migration but as well VEGF secretion is orchestrated by β2 integrins and influences tumor fate [310]. In this regard, CD11b^−/−^ but not CD11a^−/−^ mice inoculated with B16F10 melanoma or RM1 prostate cancer cells displayed attenuated tumor neovascularization as compared to WT mice. One reason for this is an impaired infiltration of tumor tissue with PMNs and macrophages, which secrete VEGF needed for vessel development. In addition, CD11b^−/−^ PMNs were characterized by markedly reduced degranulation and VEGF secretion upon TNF-α stimulation [310,311].

#### 5.3.3. Tumor-Specific Immune Responses

As discussed above, MAC-1/CR3 and CR4 confer uptake of complement-opsonized material, and MAC-1 is involved in FcR-mediated internalization of antibody-opsonized cells. As a consequence, anti-melanoma antibody treatment for induction of ADCC (antibody-dependent cytotoxicity) in a melanoma metastasis model [312] was much less effective in CD11b^−/−^ mice as in WT animals [313]. Besides complement-opsonized material, MAC-1 (CR3) and, to lower extent, CD11c/CD18 (CR4) bind a variety of soluble ligands, many of which can be found within the TME (see Section 2.4). Interaction of tumor-associated ligands with β2 integrins and the resulting immunoregulation is not well defined yet. For example, apoptotic bodies derived from tumor cells are taken up via MAC-1 (in addition to CD36) by DC, resulting in tolerance induction [126]. Thus, endocytosis of tumor-derived material and the resulting immunological response are both regulated by β2 integrins.

#### 5.3.4. Interaction with Tumor Cells

The adhesive properties of β2 integrins have been controversially described in the context of tumor growth control. In case of LFA-1, it has been shown that on one hand it is an essential modulator of the IS between an NK cell or CTL and a tumor cell and hence is responsible for both adhesion and targeted release of the cytotoxic granules that kill the tumor cell [314,315,316,317]. On the other hand, it has been reported that LFA-1 and MAC-1 mediate adhesion of PMN to ICAM-1-expressing melanoma cells, allowing tandem migration of tumor cells, and thereby their extravasation [318,319]. Thus, binding of a leukocyte via β2 integrins to a tumor cell may lead both to eradication of the tumor or to its metastasis.

#### 5.3.5. Leukemia

In chronic lymphatic leukemia (CLL) overall CD18 expression by malignant leukocytes is lower in comparison to healthy cells [320], and a functionally impaired CD18 variant (E630K) has recently been described to correlate with disease susceptibility [321]. However, in the subgroup of Trisomy 12 (tri12) harboring CLL elevated expression of LFA-1 and hence transendothelial migration was reported by several groups [322,323]. Furthermore, in CLL the adhesion of malignant B cells via LFA-1 can be disturbed due to defective inside-out signaling, involving Rap-1 GTPase affecting both β2 integrins and VLA-4 [324]. This signaling defect resulted in retention of non-functional clonal mature B cells in the blood [325,326]. Again, the subgroup of tri12 CLL differed by displaying increased levels of integrin signaling adaptors like CALDAG–GEF I, Rap-1B (Rap-1 Binding Protein), and VLA-4 [322]. Whereas Rho GTPases was required for chemokine-induced LFA-1 triggering in all CLL patients, in some, the GTPases Rac1 and Cdc42 were found dispensable [327].

#### 5.3.6. β2 Integrin Expression by Tumor Cells

Whereas LFA-1 expression is confined to leukocytes under normal conditions [6], tumor cells may express this β2 integrin as well, shown to elevate their metastatic activity [328]. The surface glykoprotein CD44 is frequently expressed by tumor cells and mediates tumor cell migration by binding collagen as a constituent of the ECM [329]. Cross-linking of CD44 on tumor cells was reported to induce LFA-1 and VLA-4 expression [330]. Both receptors equally mediate transendothelial tumor cell migration as deduced from the finding that antibody-mediated blockade of either receptor did not affect transendothelial tumor cell migration but only their combined inhibition.

In general, even though β2 integrins are required for any innate/adaptive immune response, tumor infiltration by leukocytes as mediated by LFA-1 and MAC-1 supports growth of an established tumor that already communicates with an environment in order to induce tolerance. A deeper understanding of the role of β2 integrins in the TME, especially with regard to their potential function in regulatory immune cells, may allow to develop new therapeutic strategies targeting specific β2 integrins in a cell type-specific manner.

## 6. β2 Integrins as Therapeutic Targets

In the last decades, β2 integrins have received much attention as therapeutic targets mostly for treatment of autoimmune conditions. Integrin antagonists, such as a humanized CD11a blocking antibody (Efalizumab), have been developed to inhibit LFA-1 activity and thereby to treat psoriasis [335]. However, Efalizumab was withdrawn from the market in 2009 due to JC virus reactivation in some patients [336,337]. This adverse effect was caused most likely by deficient leukocyte migration and subsequent immunodeficiency. Similar side effects, although less severe, were reported in patients treated with Natalizunamb, an antibody specific for α4 integrin [338,339]. This antibody binds α4β1 and α4β7 and has been successfully used to treat patients suffering from Morbus Crohn and MS [340,341]. The pharmacological drug BMS-587101 has been developed to selectively block CD11a and was intended to cause less severe side effects as monoclonal antibodies [342]. BMS-587101 was reported to effectively reduce lung inflammation and joint destruction in a murine RA model [343] and attenuated transplant rejection in a mouse model [342] but was not further developed. Currently, no active clinical trials with antibodies or antagonists to β2 Integrins are listed on clinicaltrials.gov.

Leukadherin-1 (LA-1), selectively activates MAC-1 and increases cell adhesion to ICAM [344]. It has been noted that LA-1 suppresses innate inflammatory signaling in human NK cells [130]. In contrast to LFA-1, As outlined above, MAC-1 not only mediates leukocyte migration, but as well contributes to tolerogenic signaling. NK cells pre-treated with LA-1 displayed less STAT5 phosphorylation in response to IL-12, and consequently reduced secretion of TNF-α and IFN-γ [130]. LA-1 has been successfully used to prevent inflammation in hypoxia-induced lung injury in rats [345] and in an autoimmune nephritis mouse model [346,347]. Since myeloid cells express both LFA-1 and MAC-1 that bind other (immune) cells via ICAM, specific blockade of either β2 integrin may be compensated by the other. Therefore, as an alternative approach, ICAM-1 blocking antibodies have been developed. A study on patients with early RA showed benefits of an ICAM-1 blocking antibody [348,349]. Unfortunately, side effects restricted further testing [348].

## 7. Concluding Remarks

β2 integrins strongly contribute to the functional activity of any type of immune cell analyzed so far, comprising regulation of innate immune functions as the recognition of pathogens, infiltration of inflamed tissue, and pathogen killing, as well as adaptive immune functions, including antigen uptake, and stimulation/polarization of T cells and B cells to induce pathogen-specific immune responses. However, triggering of β2 integrins on APC may also confer tolerance and may thus contribute to impaired clearance of pathogens and tumor development, which in turn promotes the induction/expansion of regulatory immune cells. Regarding the latter, intriguingly, the role of β2 integrins for the immunosuppressive activity and the crosstalk of regulatory immune cells, namely Treg, MDSC, and TAM, has scarcely been addressed yet. However, this knowledge is crucial to develop tailored intervention strategies that counteract the immune-inhibitory activity of regulatory immune cells.

By now, the pathophysiological role of ß2 integrins has been studied largely in various mouse models with a constitutive knock-out of either an individual α or the common β subunit. However, in all of these mouse models it is difficult to delineate at the cellular level in vivo whether any functional alteration is the consequence of an intrinsic defect or a result of crosstalk with other immune cells deficient for β2 integrins as well. To overcome these limits, mouse models that enable cell type-specific deletion of β2 integrins need to be established.

So far, β2 integrin-focused attempts to treat (autoimmune) diseases aimed to modulate the activation state of a given β2 integrin systemically. However, β2 integrins may exert contrary functions in a cell type-specific manner, e.g., limit T cell activation when expressed on APC (LFA-1, MAC-1), but be necessary for T cell activation (LFA-1) when expressed on T cells. Hence, multi-functionalized nano-therapeutics that co-deliver a cell type-targeting and a β2 integrin-modulating moiety may provide a suitable mean to overcome this obstacle.

## Figures and Tables

**Table 1:** Role of β2 integrins in autoimmune diseases.

Sub-Unit	Cell Type	Species ^1^	Disease Model, Immune State	Observations	Reference
CD18	-	h	LAD	LAD patients suffer from intestinal colitis, periodontitis, Type 1 Diabetes, autoimmune cytopenia	[166,252,253]
		m	LAD	Chronic dermatitis and splenomegaly	[177,179]
CD11a	T cell	m	EAE	Pro- or anti-inflammatory functions, depending on the experimental setup	[258,276,283]
		h	Systemic sclerosis	Expression correlates with severity of disease	[264,284]
		h	Psoriasis	Blockade with Efalizumab induces T cell hyporesponsiveness and inhibits psoriasis pathogenesis	[266]
		h	Autoimmune thrombocytopenia	High expression positively correlates with autoimmune thrombocytopenia pathogenesis	[189]
		h	SLE	Expression correlates with SLE severity	[193]
		h	RA	Expression correlates with RA pathogenesis	[285,286]
		m		Expression is essential for CIA development	[287]
CD11b	DC	m	RA	Controls balance between Th17 and Treg via IL-6	[255]
	APC	m	Peripheral tolerance	Required for establishment of orally induced peripheral tolerance (suppresses IL-6/IL-17 induction)	[249,288]
		h	RA, SLE	Polymorphisms predispose for SLE and RA	[193,276,289,290]
		m	SLE	Activation suppresses autoimmunity	[193]
	DC	m	EAE	CD11b^+^ DC accumulate in CNS of MOG-immunized mice and present with tolerogenic phenotype (IL-10 and TGF-β secretion)	[291]
	T cells	m		CD11b^+^ TC are required for the EAE development	[269,292]
	B cell	m	Experimental autoimmune hepatitis	CD11b^+^ B cells suppress T cell response by inhibiting TCR signaling	[189]
	-	h	Psoriasis	Frequency of CD11b^+^ cells in lesions correlates with MPO activity. CD11b expression in PMN/macrophages is elevated in pustular psoriasis.	[293,294,295]
	PMN	m	Bullous pemphigoid	CD11b is required for skin infiltration by PMN and inflammation development in anti-BP180 antibody-injected mice	[275]
CD11c	DC	m	Autoantibody production	cholesterol accumulation in DC contributes to autoimmune processes	[296]
CD11d	-	h/m	Obesity	CD11d expression is elevated in white adipose tissue of obese humans/mice	[297]

^1^: h: human, m: mouse.

**Table 2:** The role of β2 integrins in tumor development.

Subunit	Cell Type	Species	Tumor Model	Observations	Reference
CD18	S100A8^+^ myeloid cells	m	Lewis lung carcinoma (LLC) and MC38 colon adenocarcinoma	Tumors growing in CD18^hypo^, but not in CD11b-deficient mice were more sensitive to irradiation in comparison to WT mice	[309]
	B cell	h	CLL	CD18 variant (E630K) enhances CLL susceptibility	[321,331]
CD11a	NK, TC	h	-	Establishment of an intercellular synapse with cancer cell and targeted release of granules depend on LFA-1	[331,332,333]
	B cell	h	CLL	Impaired motility and accumulation in the blood due to defective Rap-1 GTPase signaling/diminished CD18 expression	[320,324]
CD11aCD11b	PMN	m	Melanoma	ICAM-1 expressing melanoma cells bind PMN via LFA-1/MAC-1, and thus are carried across the vasculature forming metastasis	[319]
CD11b	PMN	m	B16F10 Melanoma, RM1 prostate cancer	CD11b^−/−^ PMN fail to infiltrate tumor tissue and secrete VEGF needed for the neovascularization at lower extent	[310]
		m	spontaneous intestinal adenoma	CD11b^−/−^ myeloid cell infiltrate tumor mass at low extent, associated with diminished tumor growth and impaired Wnt/β-catenin activity in the tumor	[305]
		m	Squamous cell carcinoma xenografts	Systemic application of CD11b blocking antibody increased anti-tumor response after radiation	[309]
		h	Gastric cancer	Extent of CD11b^+^ cell infiltration correlated with tumor size, venous invasion, lymph node metastasis, general metastasis stage and FoxP3^+^ cell infiltration	[307]
		h	Epithelial ovarian cancer (EOC)	*de novo* expression of MAC-1 on EOC cell lines	[334]
		m	Melanoma	MAC-1 is essential for antibody-mediated antitumor responses	[313]
CD11c	APC	h	Gastric cancer	CD11c^+^ cell tumor infiltration correlated with tumor size.	[307]
CD11d	Macro-phage	m		Upregulation of CD11d/CD18 surface expression by myeloid cells is associated with their accumulation at the inflammation site and chronification of inflammation	[67]

## Abbreviations

ADAP	Fyn-binding protein
ADCC	Antibody-dependent cytotoxicity
ADMIDAS	Adjacent to metal-ion-dependent-adhesion-site
AP-1	Activator protein-1
APC	Antigen presenting cell
Arp2/3	actin-related protein-2/3
B cells	B lymphocytes
BCG	Bacillus Calmette–Guérin
BCL6	B cell lymphoma 6
BM-DC	Bone marrow-derived dendritic cells
BP	Bullous pemphigoid
Ca2	Calcium
CALDAG–GEF I	Ca^2+^ and diacylglycerol regulated guanine nucleotide exchange factor I
Calf-1	Calcium channel localization factor-1
Cas-L	Crk-associated substrate lymphocyte-type
CD	Cluster of differentiation
CD40L	CD40 ligand
CDC42	Cell division control protein 42 homolog
cSMAC	central supramolecular activation cluster
CIA	Collagen-induced arthritis
CLL	Chronic lymphatic leukemia
CMV	Cytomegalovirus
CTL	cytotoxic T lymphocytes
Cyr61	Cysteine-rich angiogenic inducer 61
CYTIP	Cytohesin-1 interacting protein
DC	Dendritic cells
DC-SIGN	Dendritic cell-specific ICAM-3-grabbing non-integrin
DENND1C	Differentially expressed in normal and neoplastic cells domain 1C
DOCK2	Dedicator of cytokinesis 2
EAE	Experimental autoimmune encephalomyelitis
EGF	Epidermal growth factor
EOC	Epithelial ovarian cancer
ERK	Extracellular signal-regulated kinase
ES	Embryonic stem cells
FcR	Fc receptor
FcγRIIA	Conventional type I transmembrane protein
FERMT3	Fermitin family homolog 3
Foxp3	Forkhead-Box-Protein P3
GEF	Guanine nucleotide exchange factor
h	Human
HSPC	Hematopoietic stem and progenitor cells
ICAM	Intercellular adhesion molecule
IFN-y	Interferon y
IL-6	Interleukin-6
IS	immunological synapse
ITGAM	Integrin subunit alpha M
JAB1	Jun activating binding protein-1
JAK	Janus kinase
JAM	Junctional adhesion molecule
LA-1	Leukadherin-1
LAD-I	Leukocyte adhesion deficiency type 1
Lck	Lymphocyte cell-specific protein tyrosine kinase
LIMBS	Ligand-associated metal binding site
LLC	Lewis lung carcinoma
LPS	Lipopolysaccharide
MA-ARDS	Malaria-associated acute respiratory distress syndrome
MAC-1	Macrophage antigen 1
MAPK	Mitogen activated protein kinase
MHC	major histocompatibility complex
MIDAS	Metal ion-dependent-adhesion-site
MIP	Macrophage inflammatory protein
MOG	Myelin oligodendrocyte glycoprotein
m	Mouse
MPO	Myeloperoxidase
MS	Multiple sclerosis
Mst1	Macrophage-Stimulating Protein
NFATc1	nuclear factor of activated T cells, cytoplasmic 1
NK cells	Natural killer cells
NOTCH1	Neurogenic locus notch homolog protein 1
NOX2	NADPH Oxidase 2
PI3K	Phosphoinositide 3-kinase
PIP5K1C	Phosphatidylinositol-4-phosphate 5-kinase type-1 gamma
PKC	Protein kinase C
PLC	Phospholipase C
PLD1	Phospholipase D1
PMN	Polymorphonuclear granulocytes
PRL	Phosphatase of regenerating liver 1
PSI	Plexin-semaphorin-integrin
PSGL-1	P-selectin glycoprotein ligand-1
pSMAC	Peripheral supramolecular activation cluster
PTPRG	Protein tyrosine phosphatase receptor type g
RA	Rheumatoid arthritis
Rac1	Ras-related C3 botulinum toxin substrate 1
RACK1	Receptor for activated C-kinase 1
RAGE	Receptor for advanced glycation end products
RANKL	Receptor activator of nuclear factor kappa-Β ligand
Rap-1	Ras-related protein 1
Rap1B	Rap-1 binding protein
RAPL	Regulator of adhesion and polarization enriched in lymphocytes
RhoA	Ras homolog gene family, member A
RIAM	Rap-1-GTP interacting adaptor molecule
ROS	Reactive oxygen species
SHP-1	Src homology region 2 domain-containing phosphatase 1
SKAP55,	Src kinase-associated phosphoprotein 5
SLE	Systemic lupus erythematosus
SNP	Single Nucleotide Polymorphism
SOCS-3	Suppressor of cytokine signaling 3
SPTAN1	Spectrin alpha, non-erythrocytic 1
STAT	Signal transducers and activators of transcription
STK4	Serine/threonine protein kinase 4
SYK	Spleen tyrosine kinase
T cells	T lymphocytes
TCR	T cell receptor
TEM	Transmission electron microscopy
TGLN2	Taglin 2
Thy-1	Thymus cell antigen 1
TLR	Toll-like Receptor
TME	Tumor microenvironment
TNF-α	Tumor necrosis factor alpha
Treg	Regulatory T cells
Tri12	Trisomy 12
VAV1	Vav Guanine Nucleotide Exchange Factor 1
VCAM-1	Vascular cell adhesion protein 1
VEGF	Vascular endothelial growth factor
WASp	Wiskott-Aldrich syndrome protein
WBC	White blood cells
WT	Wild type
γ/δ T cells	Gamma/delta T cells
